# Effects of RNA methylation on Tumor angiogenesis and cancer progression

**DOI:** 10.1186/s12943-023-01879-8

**Published:** 2023-12-06

**Authors:** Mingyu Han, Haifeng Sun, Quanbo Zhou, Jinbo Liu, Junhong Hu, Weitang Yuan, Zhenqiang Sun

**Affiliations:** 1 Department of Colorectal Surgery, The First Affiliated Hospital of Zhengzhou University, Zhengzhou, 450001 China; 2 Henan Institute of Interconnected Intelligent Health Management, The First Affiliated Hospital of Zhengzhou University, Zhengzhou, 450052 China

**Keywords:** RNA methylation, Tumor angiogenesis, Cancer progression, MiRNA

## Abstract

Tumor angiogenesis plays vital roles in the growth and metastasis of cancer. RNA methylation is one of the most common modifications and is widely observed in eukaryotes and prokaryotes. Accumulating studies have revealed that RNA methylation affects the occurrence and development of various tumors. In recent years, RNA methylation has been shown to play an important role in regulating tumor angiogenesis. In this review, we mainly elucidate the mechanisms and functions of RNA methylation on angiogenesis and progression in several cancers. We then shed light on the role of RNA methylation-associated factors and pathways in tumor angiogenesis. Finally, we describe the role of RNA methylation as potential biomarker and novel therapeutic target.

## Introduction

Cancers represent complex ecosystems comprising tumor cells and a multitude of non-cancerous cells, embedded in an altered extracellular matrix [1]. Angiogenesis is the process by which blood vessels are formed through the proliferation and migration of endothelial cells on the basis of original blood vessels; indeed, the vasculature is much more than a passive, homogeneous conduit for oxygen and nutrient supply [2]. Under physiological conditions, angiogenesis plays significant roles in wound healing, bone repair and regeneration, and the transportation of substances that are needed by the human body [3–5]. Moreover, angiogenesis acts an essential role in tumor metastasis and expansion, atherogenesis and inflammatory diseases under pathological conditions [6–9]. In most cases, the progression of a small mass of cancerous cells to a life-threatening tumor depends upon the initiation of angiogenesis and involves the dysregulation of the angiogenic balance [10].

**Fig. 1:**
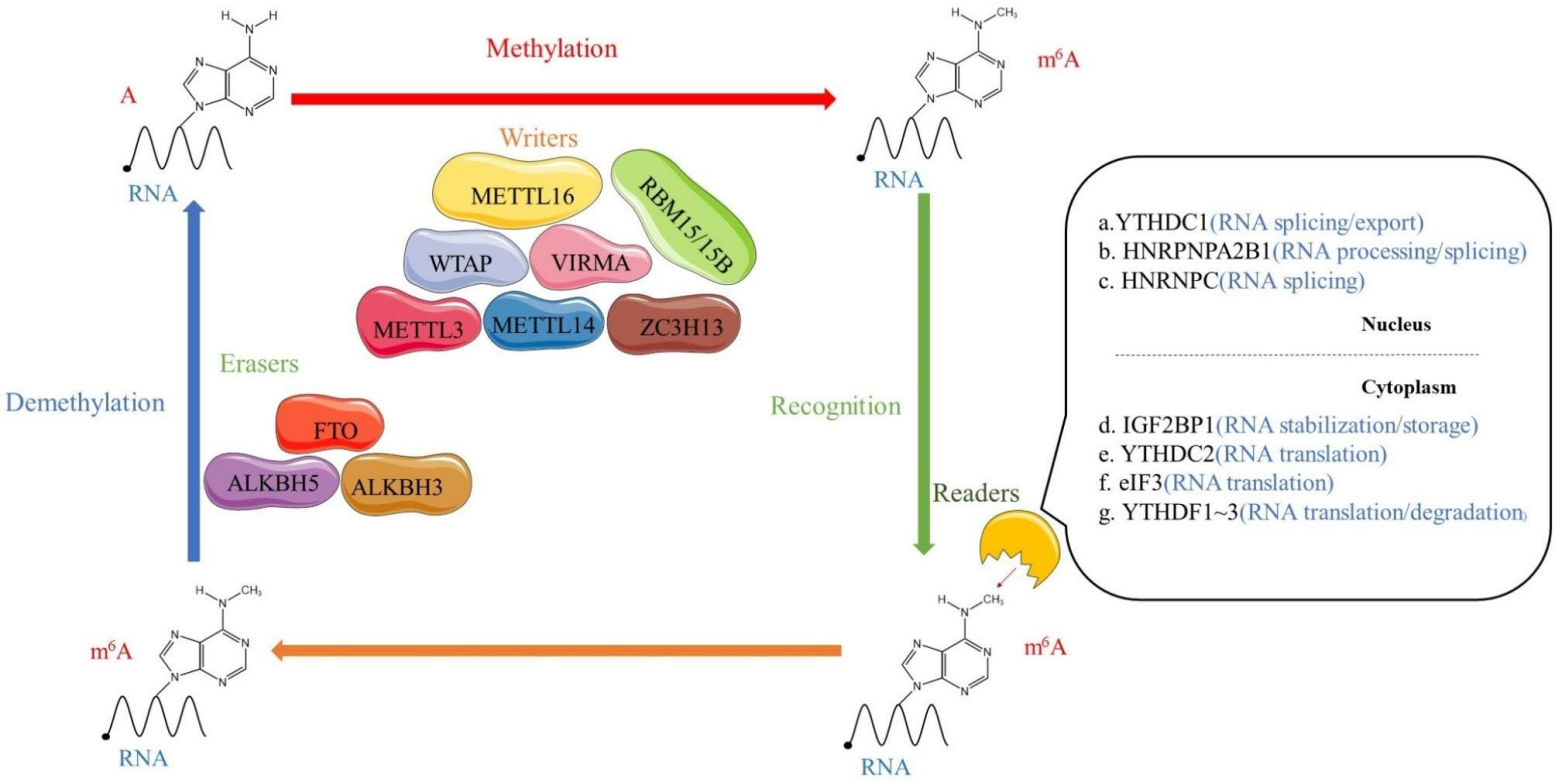
The molecular mechanism of m6A modification. m6A is installed by “writers” (METTL3/14, WTAP, RBM15/15B, VIRMA, METTL16 and ZC3H13), removed by “erasers” (FTO, ALKBH5, and ALKBH3, termed “erasers”), and recognized by “readers” (YTHDC1/2, YTHDF1/2/3, IGF2BP1/2/3, HNRPNPA2B1, HNRNPC, and eIF3, termed “readers”)

After the discovery of the first RNA demethylase (fat mass and obesity-associated protein, FTO) more than 10 years ago, researchers gradually elucidated that RNA methylation is a reversible process [11, 12]. This discovery also made RNA methylation research a point focus for scholars. With the development of RNA methylation immunoprecipitation sequencing technology, an increasing number of RNA methylation sites have been identified [13, 14]. RNA methylation participates in the regulation of RNA splicing [15–17] and protein synthesis [18, 19]. To date, over 100 different types of RNA modifications have been identified [20], and N6-methyladenosine (m6A) is the most common RNA modification that is observed in mRNAs [21]. **(**Fig. 1**)**. In human cancer, abnormal m6A modification has been reported to affect tumor proliferation, migration and invasion [22].

At present, with increasing research on tumor angiogenesis, researchers have come to understand that there is a relationship between RNA methylation and tumor angiogenesis [23, 24]. Currently, the association between RNA methylation and tumor angiogenesis and the effect of this association on tumor progression remain unclear. In this review, we summarize the role of RNA methylation in tumor angiogenesis and describe the effects of RNA methylation on some types of tumors and the mechanisms underlying their functions. Furthermore, we propose that RNA methylation has clinical value as a cancer biomarker and therapeutic target.

## Function and mechanism of RNA methylation in regulating Tumor angiogenesis

In 1971, Folkman first suggested that angiogenesis was required for the development and growth of solid tumors beyond the size of 1‑2 mm^3^ [25]. In addition, tumor tissues are characterized by high angiogenic capacity. The RhoA and PI3K-Akt pathways, which are common signal transduction pathways, play important roles in the development and progression of cancer, especially in the regulation of TGFβ induced epithelial mesenchymal transition (EMT), tumor progression and angiogenesis [26]. TGFβ, which is the target gene of methyltransferase-like protein 14(METTL14), is regulated by the upregulation or knockout of METTL14. METTL14 modifies TGFβ through the RhoA and PI3K-Akt pathways, which are considered to be related to tumor angiogenesis and tumor progression [27]. The Wnt/Fzd/β- catenin signalling pathway is a common pathway through which many factors [28] and various enzyme proteins to regulate tumor angiogenesis [29, 30]. For example, the BCL9 protein, which is a transcriptional Wnt/β-catenin cofactor, is the angiogenesis promoting element of the Wnt pathway in colorectal cancer (CRC) [31]. RNA methylation recognition factors affect the Wnt/β-catenin pathway in an m6A dependent manner [32], which may ultimately affect on tumor angiogenesis. The HIF-1α/VEGF/VEGFR pathway is currently considered to be related to angiogenesis and tumor progression in a variety of tumors [33–35]. Among the components of this pathway, the vascular endothelial growth factor (VEGF) family is considered to be an important regulator of tumor angiogenesis, and VEGF and its receptor VEGFR are involved in the regulation of tumor angiogenesis and tumor growth [36]. The expression of VEGF and hypoxia inducible factor HIF-1 α have the interwovenness; the latter binds to hypoxia response elements in the promoter of the VEGF gene to participate in the regulation of VEGF expression [37]. RNA methylation molecules, such as methyltransferase-like protein 3(METTL3) and insulin growth factor-2 binding protein 3(IGF2BP3), have been shown to potentially influence the expression of HIF-1α via RNA methylation [38, 39]. Therefore, RNA methylation may affect tumor angiogenesis and tumor progression through the HIF-1 α/ VEGF /VEGFR pathway.

TGFβ promotes tumor growth and metastasis by inducing factors associated with angiogenesis and promoting EMT [40]. Furthermore, RNA methylation plays an important role in the regulation of TGFβ, which has been proven to play a key role in tumor progression. In support of this finding, hypoxia (which is known to promote cancer progression, angiogenesis and metastasis) changes the levels of m6A writers, erasers and readers, resulting in a decrease in m6A levels and an increase in TGFβ1 expression in cancer cells. Some studies have noted that the expression of PDGF ( TGFβ important effectors of tumor progression) [41] and high mobility group A2 (HMGA2) [42] is regulated by changes in m6A levels. In addition, the expression of connective tissue growth factor (CTGF) [43], which is a direct target of angiogenesis-promoting medium and the TGFβ signalling pathway, is significantly reduced in METTL14/ALKBH5-silenced cancer cells [27].

CD34 is another tumor marker that is involved in angiogenesis and has been used as a quantitative indicator of microvessel density (MVD) [44]. An association was observed between low FTO expression and high CD34 expression in intrahepatic cholangiocarcinoma (ICC) [45]. The upregulation or downregulation of FTO-related genes may affect the expression of some relevant oncogenes in tumor cells by affecting mRNA methylation; for example, chemokine CCL19 expression is downregulated in tumor tissues after the knockdown of FTO genes [45]. The chemokine CCL19 has been demonstrated to inhibit angiogenesis in colorectal cancer through a CC-chemokine receptor 7 (CCR7)-dependent manner [46]. These results suggest that FTO inhibition might promote tumor angiogenesis [45]. METTL14 affects tumor angiogenesis in a manner that is related t to TRAF1 [47]. In addition, METTL3, YTHDF3 and IGF2BP3 affect tumor angiogenesis by regulating the expression of VEGF.

## Regulatory effects of m6A modifiers on angiogenesis in cancers

The m6A modification needs to be catalysed by the methyltransferase complex, which mainly consists of METTL3 and METTL14 [48, 49] and their cofactors, such as wilms tumor 1 associated protein (WTAP) [50], RNA-binding motif 15/15B (RBM15/15B) [51], Cbl proto-oncogene like 1 (CBLL1) [52], Vir-like m6 A methyltransferase associated (VIRMA) [53], zinc finger CCCH-type containing 13 (ZC3H13) [54]and METTL 16 [55]. These factors are considered to be the writers and play an essential role in RNA methylation.

Among these writers, METTL3 mediates a methylation process that is involved in transcriptional regulation, protein, phosphorylation and tumor angiogenesis in tumor tissues [56]. Conversely, METTL3 inhibition or selective knockdown has been proven to reduce the expression of specific targets that are related to tumor angiogenesis, thereby significantly repressing tumor angiogenesis [57–60]. Similar to METTL3, METTL14 has been proven to participate in the regulation of RNA methylation. It has been suggested that silencing METTL14 may inhibit tumor angiogenesis [27, 47, 61]. For example, studies have reported that tumor necrosis factor receptor (TNFR) associated factor 1 (TRAF1), which is a signalling adaptor that was first recognized as a part of the TNFR2 signalling complex, plays various roles in human disease [62, 63]. TRAF1 overexpression significantly enhances angiogenesis, whereas reduced TRAF1 expression inhibits angiogenesis [47]. Both effects are mediated via the AKT/mTOR/HIF1a/VEGFA pathway. METTL14-mediated m6A modification promotes the stability of the TRAF1 mRNA in an IGF2BP2-dependent manner, thereby significantly promoting tumor angiogenesis [47].

RNA methylation is a dynamic and reversible modification process, and the FTO gene and alkb homologue 5 (ALKBH5) are considered to be involved in m6A demethylation modification [64]. Both of these molecules are called erasers. In addition, ALKBH3 has recently been proven to participate in m6A demethylation, especially in tRNA demethylation [65]. Screening m6 A levels by silencing the demethylase ALKBH5 inhibits cancer growth and angiogenesis. In ALKBH5-knockdown cells, the expression of some genes that are related to angiogenesis as well as cell cycle progression is decreased, which in turn inhibits angiogenesis and tumor progression [27]. FTO, which is the first m6A demethylase that was identified, belongs to the superfamily of Fe (II)-and 2-oxoglutarate-dependent dioxygenases and regulates integrin signalling pathways, inflammatory signalling pathways, epidermal growth factor receptor (EGFR) signalling pathways, angiogenesis and pyrimidine metabolism pathways [45]. When FTO is knocked down, the expression of angiogenesis inhibitors in tumor tissues is reduced, thus promoting tumor angiogenesis [45].

“Readers” are a class of variable RNA-binding proteins that have been shown to specifically select, recognize m6A sites and convey information, thus helping establish an efficient and orderly m6A regulatory network. The existing reports about readers primarily focus on three main categories. The first category includes the members of the YT521-B homology (YTH) domain family, including YTHDF1/2/3 and YTHDC1/2. These molecules have conserved m6A-binding domains that bind to RNA containing m6A, and they are the most important readers [66]; Second, the members of the heterogeneous nuclear ribonucleoprotein (HNRNP) family also play an important role in acting as readers. HNRNPA2/B1 is capable of recognizing m6A on a subset of primary microRNA (pri-miRNA) transcripts and interacts with drosha ribonuclease III (DROSHA) and DiGeorge syndrome critical region 8 (DGCR8), thus facilitating pri-miRNA processing [67]; Third, insulin-like growth factor 2 mRNA-binding proteins (IGF2BPs, including IGF2BP1/2/3) that identify m6A and promote mRNA stability and translation in an m6A-dependent manner are also involved in RNA methylation [68]. Among these proteins, knockdown of the IGF2BP3 gene inhibits hypoxia-induced angiogenesis in vivo by downregulating HIF1A, which inhibits angiogenesis mainly by reducing the expression of pro-angiogenic factor (VEGF) in tumor tissues [39, 69]. Moreover, lower YTHDF2 protein levels are significantly associated with more multinodular tumor and microvascular infiltration, higher TNM and Barcelona Clinic Liver Cancer (BCLC) staging classification, and shorter overall and recurrence-free survival [70]. Mechanistically, YTHDF2 mediates the decay of m6A-containing interleukin 11 (IL11) and serpin family E member 2 (SERPINE2) mRNAs, which are responsible for the disruption of vascular normalization. Therefore, a reduction in hypoxia-sensitive YTHDF2 could reprogram the m6 A-edited transcriptome and promote the development of hepatocellular carcinoma (HCC) [70]. YTHDF3 promotes the interaction of cancer cells with brain endothelial cells and astrocytes, blood-brain barrier extravasation, angiogenesis and growth. YTHDF3 expression promotes angiogenic brain metastasis. The vascular density in metastatic tumor tissues is significantly reduced after YTHDF3 knockdown, and the expression of VEGFA and other genes is positively correlated with the expression of YTHDF3. When both genes are simultaneously knocked down, the angiogenic ability of tumor tissues is further reduced(Table 1) [71].

**Table 1:** The functions of m6A enzymes in the angiogenesis of various cancers

Type	Molecule	Role in tumors	Tumor type	Mechanism	References
Writers	METTL3	Oncogene	Bladder cancer	Promote angiogenesis via modulating TEK and VEGF-A	[72]
		Oncogene	GC	Promote angiogenesis via stimulating m6a modification of HDGF mRNA	[58]
		Oncogene	HCC	Promote angiogenesis via modulating Hippo pathway	[57]
		Oncogene	RCC	Promote angiogenesis via modulating HIF-2α	[73]
	METTL14	Oncogene	Breast cancer	Promote angiogenesis via modulating VEGFA and RhoA and PI3K-Akt pathways	[27]
Erasers	FTO	Suppressor gene	ICC	FTO expression is inversely correlated with MVD.	[45]
	ALKBH5	Oncogene	Breast cancer	Promote angiogenesis via modulating VEGFA and RhoA and PI3K-Akt pathways	[27]
Readers	IGF2BP2	Oncogene	Lung cancer	Promote angiogenesis via modulating HNF4G/IGF2BP2/TK1 axis	[74]
	IGF2BP3	Oncogene	GC	Promote angiogenesis via enhancing HDGF mRNA stability	[58]
		Oncogene	CRC	Promote angiogenesis via modulating VEGF	[39]
		Oncogene	GBM	Promote angiogenesis via modulating PI3K/MAPK pathways	[75]
	YTHDF1	Oncogene	GC	Promote angiogenesis via modulating Wnt/β-catenin pathway	[32]
	YTHDF2	Suppressor gene	HCC	Suppress angiogenesis via downregulating IL11 and SERPINE2	[70]
	YTHDF3	Oncogene	Breast cancer	Promote angiogenesis via modulating VEGFA and EGFR	[71]

## Effects of RNA methylation on angiogenesis in different cancers

**Fig. 2:**
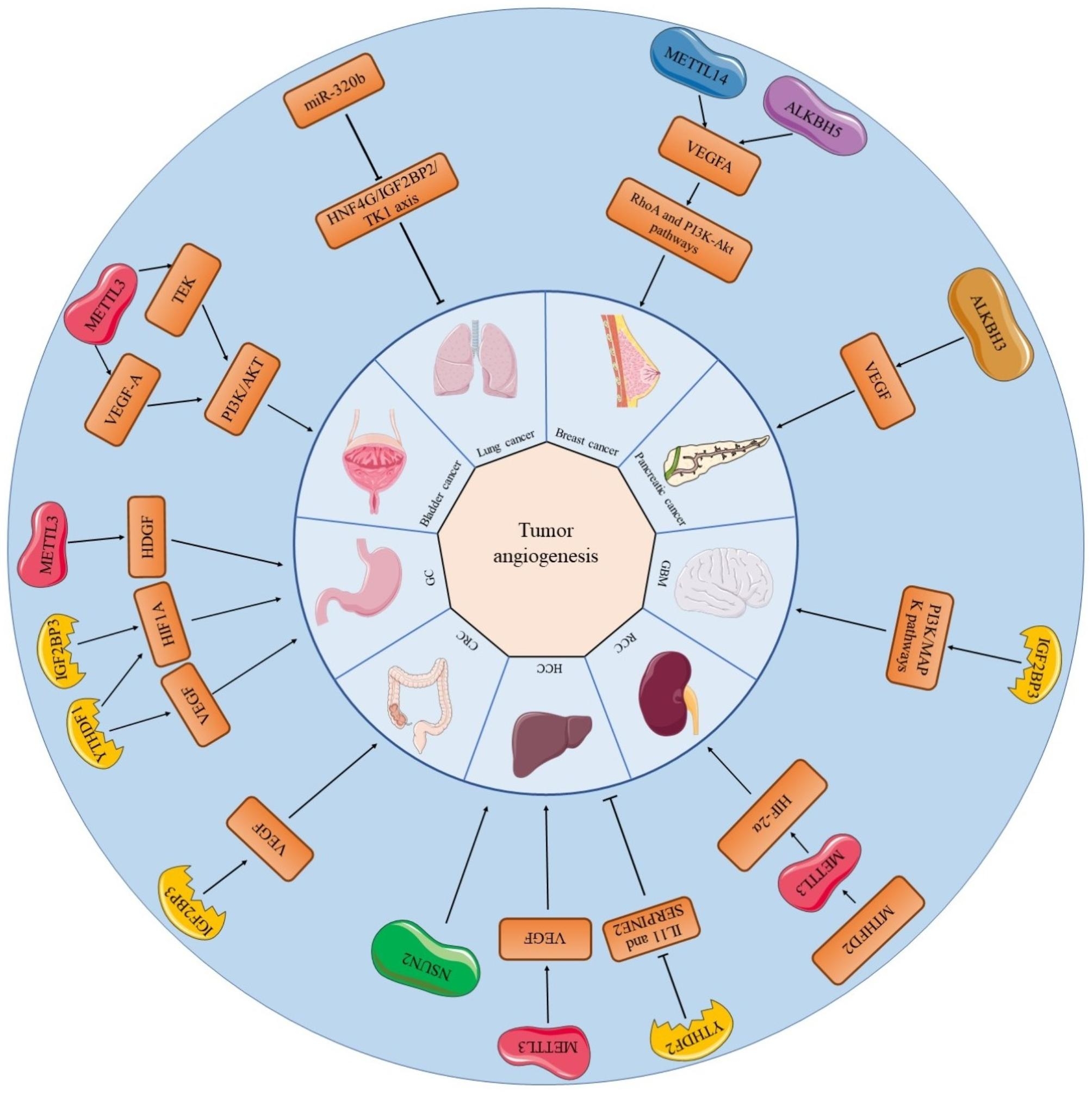
RNA methylation regulators related to angiogenesis in different cancers and their related mechanisms. Angiogenesis in some cancers is associated with more than one RNA methylation regulators. The mechanism of tumor angiogenesis varies from one tumor to another

Bladder cancer is among the top ten most common cancer types in the world, with approximately 550,000 new cases annually [76]. METTL3-mediated methylation may be involved in transcriptional regulation, protein, phosphorylation and angiogenesis in bladder cancer. For example, there are abundant m6A peaks in the TEK gene as well as VEGF-A gene, which are associated with tumor angiogenesis [77, 78]. In contrast, up- or down-regulation of METTL3 in bladder cancer cells can enhance or inhibit the transcriptions and protein expression of TEK and VEGF-A, which are components of the PI3K/AKT pathway respectively, thereby affecting the angiogenesis of bladder cancer via RNA methylation [72] **(**Fig. 2**)**.

Gastric cancer (GC) is the fifth most common cancer and the third most common cause of cancer-related death worldwide [79]. In gastric cancer, the upregulation or knockdown of METTL3 is followed by an increase or decrease respectively in m6A levels [58]. Mechanistically, P300-mediated H3K27 acetylation in the promoter region of METTL3 induces METTL3 transcription, which stimulates the m6A modification of hepatoma-derived growth factor (HDGF) mRNA; then the m6A reader IGF2BP3 directly recognizes and binds to the m6A site on HDGF mRNA and enhances HDGF mRNA stability. Secreted HDGF promotes tumor angiogenesis, while nuclear HDGF activates glucose transporter type 4 (GLUT4) and enolase 2 (ENO2) expression, which is followed by increased glycolysis in GC cells. In METTL3 overexpressing gastric cancer tumor tissues, MVD, as assessed by staining for CD31 (a marker of angiogenesis), is significantly increased compared to controls [58]. In hypoxic environments, hypoxic tumor cells undergo multiple alterations at the molecular, cellular and phenotypic levels (e.g., increased migration, invasion and angiogenesis), which contribute to their survival at primary and secondary sites or escape from unfavorable tumor environments [80, 81]. IGF2BP3 is involved in the regulation of the hypoxic responses [82, 83], which may account for the angiogenesis in cancerous tissues [39]. In gastric cancer cells that are exposed to hypoxia, deletion of IGF2BP3 suppresses VEGF expression. Studies have revealed that this effect can be achieved in gastric cancer cells by regulating HIF1A expression through IGF2BP3 binding to a specific m6A site on HIF1A mRNA [69]. HIF1A has been shown to be a major hypoxic response factor [84]. In hypoxic environment, HIF1A signalling has been proven to regulate a variety of tumorigenesis and progression, such as tumor cell survival, proliferation, metastasis, and angiogenesis [85, 86] **(**Fig. 2**)**.

Although colorectal cancer (CRC) has been studied for years, it is still one of the leading causes of cancer-related death worldwide [87]. IGF2BP3 binds to and reads the m6A recognition site in VEGF mRNA in colon cancer cells, thus regulating the expression and stability of VEGF mRNA. While VEGF is an important growth factor for tumor angiogenesis, knockdown of IGF2BP3 may inhibit angiogenesis in colon cancer by regulating the reduction of VEGF expression [39] **(**Fig. 2**)**.

Hepatocellular carcinoma (HCC) is the fourth leading cause of cancer-related death worldwide, despite the liver being the sixth most common site of primary cancer [88]. The concept of vasculogenic mimicry (VM) offers a new perspective on the supply of blood to tumors [89]. Existing studies have confirmed that VM is associated with angiogenesis in a variety of tumors, including HCC [90, 91]. Although VM has been proven to be induced by various extracellular ligands and microenvironments, VEGFa is considered to be the major inducer of this process in HCC cells [92]. The level of m6A in VEGFa-treated cells is also increased and positively correlated with the expression of the RNA methylation enzyme METTL3 in the cells. In contrast, when the expression of METTL3 was silenced, the level of m6A in HCC tissues was decreased, which affected VM [57]. In addition, METTL14 expression was significantly reduced in HCC and was significantly associated with microvascular infiltration [61]. Furthermore, NSUN2, which is the writer of 5-methylcystosine (m5C), has been proved to participate in the regulation of invasion, metastasis, and angiogenesis in HCC. [93] **(**Fig. 2**)**.

Lung cancer remains the leading cause of cancer related deaths worldwide [94]. METTL3 promotes the growth, survival and invasion of lung cancer cells, and its deletion strongly inhibits cancer cell growth, increases apoptosis, and significantly reduces the invasive ability of cancer cells [59]. In lung cancer, the anti-cancer gene miR-320b downregulates the expression of IGF2BP2 and thymidine kinase 1 (TK1), thus suppressing angiogenesis and lung cancer growth [74] **(**Fig. 2**)**.

Breast cancer is one of the most common malignancies in women throughout the world and is the major cause of most cancer-related deaths [95]. RNA methylation plays a vital role in promoting the angiogenesis and progression of breast cancer. METTL14 and ALKBH5 regulate key cell cycle- and angiogenesis-associated genes transcriptions. METTL14 or ALKBH5 overexpression leads to increased expression of target genes including VEGFA compared to vector control [27]. Angiogenesis is a major step in metastasis. The blood vessel density of metastases formed by YTHDF3 KD human breast cancer MDA-MB-231 parental cells was significantly lower than that of metastases formed by control cells. These results suggest that YTHDF3 expression facilitates angiogenic brain metastasis. Overexpression of YTHDF3 increases the invasive and angiogenic potential of cells in vitro and promotes breast cancer metastasis [71]. In addition, in breast cancer tissues, FTO overexpression was significantly correlated with tumor size, peritumor lymphovascular infiltration, and lymph node metastasis [96] **(**Fig. 2**)**.

Glioblastomas (GBMs) are heterogeneous and invariably lethal tumors [97]. In glioma cells, upregulation of IGF2BP3 expression promotes tumor proliferation, invasion, migration and angiogenesis, and is also significantly associated with lower survival rates [75] **(**Fig. 2**)**.

Renal cell carcinomas (RCCs) are kidney tumors that arise from the epithelial layer of the nephron [98]. Methylenetetrahydrofolate dehydrogenase 2 (MTHFD2) plays a critical role in controlling global m6A methylation levels, including the m6A methylation levels of HIF-2α mRNA, which results in enhanced translation of HIF-2α. In addition, VEGFA, which is related to tumor angiogenesis, is a transcript that is targeted by HIF-2α [73] **(**Fig. 2**)**.

Pancreatic cancer is a malignancy with a poor prognosis and high mortality rate [99]. PCA-1 has been shown to be identical to ALKBH3, which is the demethylase of m1A modification [100]. Previous studies indicated that PCA-1/ALKBH3 silencing reduces VEGF expression in human pancreatic cancer and that PCA-1/ALKBH3 downregulation significantly decreases the number of microvessels in tumors [101] (Fig. 2).

## Effect of RNA methylation-associated miRNAs on Tumor angiogenesis

MicroRNAs (miRNAs) are small endogenous RNAs that regulate gene-expression at the posttranscriptional level [102]. Relevant reports indicate that m6A modification participates in regulating the biogenesis of miRNA [103, 104]. Among these miRNA targets, some are involved in the regulation of tumor angiogenesis [91].

Endothelial cells(ECs) play a vital role in tumor angiogenesis [105]. METTL3 depletion in ECs decreases mature angiogenic miRNAs let-7e-5p and the miR-17-92 cluster, and increases the expression of their common target, Tsp1 (thrombospondin 1). Conversely, METTL3 increases the expression of let-7e-5p and miR-17-92 cluster and reduces protein levels of Tsp1 in ECs [106]. Therefore, miR-17-92 regulates tumor angiogenesis via m6A modification.

Previous studies have indicated that zinc ribbon domain-containing 1-antisense 1 (ZNRD1-AS1) is described as an oncogene in several types of cancer [107, 108]. ZNRD1-AS1 overexpression inhibits cell proliferation, migration and angiogenesis. YTHDC2 promotes ZNRD1-AS1 stability in malignant lung cells via m6A modification. Furthermore, the miR-942/tensin1 (TNS1) axis was demonstrated to be the downstream regulatory signaling pathway of ZNRD1-AS1. ZNRD1-AS1 promotes malignant lung cell proliferation, migration, and angiogenesis via the miR-942/TNS1 axis and is positively regulated by the m6A reader YTHDC2 [109].

The expression of circ-CCT3 is remarkably upregulated in HCC and associated with poor prognosis. Functionally and mechanistically, circ-CCT3 promotes the angiogenesis of HUVEC by the sponge activity on miR-378a-3p and improve FLT1 expression, indicating its potential tumor promoter role in liver cancer development. Furthermore, the m6 A regulator (ALKBH5 and METTL3) could bind and regulate m6 A-modification of circ-CCT3. In summary, Circ-CCT3 promotes angiogenesis via miR-378a-3p-FLT1 axis in HCC and is under m A-modification mediated by ALKBH5 and METTL3 [110].

In METTL14-deficient cells, the expression of miR126 is reduced, since it is downstream target of METTL14 and regulated in an m6A-dependent manner, and it is involved in metastasis and vascular infiltration of HCC. Through the study, miR-126 was found to be significantly reduced in the vascular infiltration positive group and slightly reduced in the liver transplantation recurrence group [61].

Relevant research indicates that knocking down the expression of METTL3 reduces the expression of miR-143-3p in cells. Conversely, when METTL3 was overexpressed, it can in turn promote the expression of miR-143-3p in cells. Angiogenesis is significantly increased in cultures that are transfected with miR-143-3p, and overexpression of miR-143-3p can increase VEGFA expression in lung cancer cells, thereby regulating angiogenesis in lung cancer tissues [111]. In addition, the expression of regulators associated with tumor metastasis or angiogenesis, such as Vasohibin-1 (VASH1), is also affected in cells transfected with miR-143-3p. One study showed that miR-143-3p might be involved in negatively regulating the expression of VASH1, which in turn was involved in promoting cancer cell dissemination and angiogenesis [112].

In CRC tissues, circ3823 is significantly highly expressed, and m6A modification has been proven to be involved in regulating its degradation. It has been demonstrated that circ3823 may promote the proliferation, metastasis and angiogenesis of colorectal cancer through the circ3823/miR-30c-5p/TCF7 axis [113]. Another study showed that the overexpression of miR-320b can inhibit cell invasion and angiogenesis in vitro and suppress angiogenesis and tumor growth in vivo. Hepatocyte nuclear factor 4 gamma (HNF4G) is a direct target gene of miR-320b, which has been proven to upregulate IGF2BP2 expression [74]. Furthermore, it has been reported that IGF2BP2 can recognize the m6A modification of the TK1 gene to increase TK1 expression [68], thus promoting angiogenesis.

In summary, these findings suggest that various miRNAs exert effects that regulate tumor angiogenesis via mechanisms that are related to RNA methylation. This discovery may provide distinguished indicators and drug intervention targets for cancer treatment.

## Regulation of Tumor angiogenesis by Hippo/YAP1 pathway-related RNA methylation

In general, yes-associated protein (YAP) and transcriptional co-activator with PDZ-binding motif (TAZ) are inhibited by the Hippo pathway in normal quiescent adherent cells, and this process occurs in the cytoplasm [114]. The Hippo pathway is an important pathway that plays a crucial role in the malignant progression of cancer [115]. Relevant studies have indicated that various types of cancers, including breast, lung, pancreatic, liver, colorectal, gastric cancer and glioma, are accompanied by increased YAP/TAZ levels and activity [116–123]. Furthermore, numerous studies have noted that the Hippo/YAP1 pathway is closely related to RNA methylation and proteins associated with RNA methylation, and the methylation of RNAs that are associated with this pathway may regulate the pathway [56, 123–126]. In addition, recent studies have proposed that the Hippo/YAP1 pathway is inextricably linked to tumor angiogenesis [127, 128]. There is a question: does RNA methylation participate in regulating tumor angiogenesis through the Hippo/YAP1 pathway? The answer is yes. Currently, numerous relevant studies indicate that Hippo/YAP1 pathway-related RNA methylation plays a regulatory role in tumor angiogenesis. The role of the Hippo pathway in m6A-mediated VM formation was investigated, and the level of m6A modification of YAP1 mRNA was significantly reduced. This study indicates that YAP1 promotes tumor vasculature formation and malignant progression in an m6A-dependent manner in vitro. YAP1 is suppressed by the downregulation of METTL3 after a decrease in m6A, and upregulated by the overexpression of ALKBH5 (an important m6A demethylase) and the induction of VEGFa after an increase in m6A. Furthermore, m6A has been proven to alter the expression of the YAP1 protein by increasing translation efficiency, which may trigger the degradation of YAP1 pre-mRNA and the degradation of mature mRNA in HCC [57]. Additionally, accumulating studies have confirmed that the m6A demethylase ALKBH5 inhibits tumor growth, metastasis, invasion, and tumor angiogenesis in NSCLC by reducing YTHDFs-mediated YAP1 expression and inhibiting miR-107/LATS2-mediated YAP1 activity. This mechanism indicates that ALKBH5 may reduce the m6A level of YAP1 precursor mRNA in NSCLC, thus affecting the Hippo/YAP1 pathway [124].

## Effects of RNA methylation on the blood-brain barrier and angiogenesis associated with Tumor metastasis

The blood–brain barrier (BBB) is a continuous endothelial membrane within brain microvessels that are composed of sealed cell-to-cell contacts, and is sheathed by mural vascular cells and perivascular astrocyte end-feet [129]. Currently, the main sources of metastatic brain cancer are lung cancer and breast cancer [130]. Tumor cells that have entered the bloodstream need to penetrate the BBB to gain access to the brain parenchyma [131]. Therefore, the development of brain metastatic carcinoma is usually associated with abnormalities in the blood-brain barrier, a process that is dependent on abnormal angiogenesis. For example, miR-143-3p expression is upregulated in brain metastatic cancer tissues compared to primary cancer tissues [111]. Inhibition of vasopressor-1 (VASH1) expression by targeting the three binding sites that control its 3’UTR enhances the invasiveness and angiogenesis of lung cancer in an in vitro BBB model. The m6A methyltransferase METTL3 increases splicing of the precursor miR-143-3p to promote its biogenesis. Conversely, transfection with miR-143-3p inhibitors blocks angiogenesis as well as VEGFA expression in tumor tissues [111].

YTHDF3 contains a C-terminal YTH structural domain that specifically binds to m6A-containing RNAs. It has been suggested that overexpression of the m6A reader YTHDF3 is clinically associated with brain metastasis in breast cancer patients. YTHDF3 promotes the interaction of cancer cells with brain endothelial cells and astrocytes, blood-brain barrier extravasation, angiogenesis and growth. Mechanistically, YTHDF3 enhanced the translation of m6A-enriched transcripts of ST6GALNAC5, GJA1 and EGFR, all of which are associated with brain metastasis. Notably, all of these key brain transfer gene transcripts are enriched in the m6A peak. This study also demonstrates the role of YTHDF3 in facilitating the translation of key brain transfer transcripts and that the process is dependent on the binding of YTHDF3 to m6A-modified mRNAs [71].

## Effects of RNA methylation on Tumor angiogenesis and Tumor drug resistance

Drug resistance remains a major challenge to the curative treatment of various cancers [132]. Accumulating evidence has revealed that RNA methylation regulates multiple anticancer drug resistance, including drug transport and metabolism, target receptors, cancer stemness, DNA damage repair and cell death [21, 133–136]. In addition, the variation contributes to drug resistance by regulating DNA damage repair, downstream adaptive responses (apoptosis, autophagy, and oncogenic bypass signaling), cell stemness, the tumor immune microenvironment, and exosomal non-coding RNAs [137].

In previous studies, FOXO3 was identified as a key transcription factor in multiple oncogenic pathways, and it is involved in regulating cell cycle arrest [138], apoptosis [139] and autophagy [140]. Sorafenib is a multitarget drug that inhibits cancer cell proliferation and angiogenesis [141]. Recent studies have noted that FOXO3 is a direct target of METTL3 in HCC cells under intratumor environmental conditions. Silencing METTL3 at the RNA and protein levels significantly reduces the expression of FOXO3. This study also indicates that METTL3-mediated m6A modification promotes FOXO3 RNA stability in a YTHDF1-dependent manner. The reduction in METTL3-m6A function enhances autophagy and angiogenesis in HCC through the METTL3/FOXO3 axis in vivo, thus enhancing sorafenib resistance [142].

EGFR plays a significant role in angiogenesis and drug resistance in CRC [143]. The anti-EGFR antibody cetuximab has been used for CRC therapy for several years [144]. As mentioned previously, FTO and YTHDF3 were proved to influence on the expression of EGFR [45, 71]. Additionally, the m6A reader hnRNPA2B1 plays a vital role in the transcriptional activity of Wnt signalling in CRC via regulation of TCF7L2 mRNA stability, which may contribute to cetuximab resistance [145]. METTL3 enhances the translation efficiency of EGFR, followed by rebound activation of RAF/MEK/ERK, resulting in acquired PLX4032 resistance in melanoma [146]. Besides, YTHDF1 and YTHDF2 affect cancer by binding to the m6A sites in the 3′-UTR of EGFR transcription and contributed to aberrant activities of downstream signal pathways [147, 148].

As a multitarget receptor tyrosine kinase (RTK) inhibitor, sunitinib plays an important role in the treatment of RCC [149]. Previous studies revealed that TRAF1 overexpression promotes sunitinib resistance by regulating apoptosis and angiogenic pathways in a METTL14-dependent manner [47].

Protein arginine methyltransferase 5 (PRMT5) is highly expressed in multiple types of cancer and reported to decreased the breast cancer cell response to doxorubicin [150, 151]. Doxorubicin treatment markedly induces RNA m6A methylation in breast cancer cells and tumor tissues. However, PRMT5 inhibits doxorubicin-induced RNA m6A methylation by enhancing the nuclear translocation of ALKBH5 to the partner AlkB homolog 7 (ALKBH7). ALKBH5 removes the m6A methylation from BRCA1 mRNA and maintains the stability and function of BRCA1, which reduces the sensitivity of cells to doxorubicin [152].

These findings reveal that RNA methylation has immense potential for regulating drug resistance.

## The potential of RNA methylation in clinical applications

An increasing number of studies have revealed that RNA methylation is very common in cancer [20]. To illustrate, METTL3, which is an RNA methylation enzyme, is inextricably linked to the process of RNA methylation in tumor cells. Currently, an increasing number of studies point to METTL3 as a biomarker that has a definite relationship with tumor prognosis, metastasis, diagnosis, and drug resistance [153–157]. For example, it has been demonstrated that the mRNA levels of METTL3 are significantly overexpressed in GC tissues and that patients with high METTL3 expression have a shorter median survival time. In addition, elevated expression of METTL3 was significantly associated with TNM stage and vessel invasion of tumors in gastric cancer patients [154]. Moreover, a relevant study also demonstrates that METTL3 expression is an independent prognostic factor and effective predictor in human patients with GC [58].

Moreover, relevant studies indicate that IGF2BP3 is not only a prognostic biomarker but also a diagnostic molecule [158]. An increasing number of studies have shown that the expression of IGF2BP3, as determined by immunohistochemistry, has prognostic significance in a number of cancers, including RCC, CRC, oophoroma, mammary carcinoma, pancreatic ductal adenocarcinoma, cervical carcinoma, and GBM [75, 159–164]. IGF2BP3 has also been shown to be associated with aggressive and advanced carcinomas [165–170]. For example, IGF2BP3 tends to exert oncogenic effects in most malignant gliomas in which IGF2BP3 is overexpressed and plays a role in promoting proliferation, invasion, migration, angiogenesis, and chemoresistance. In addition, IGF2BP3 was specifically highly expressed in glioma cells, enhancing its role as a diagnostic marker. Moreover, the median survival rate of patients that overexpress this protein was lower, indicating its potential as a prognostic indicator for disease evolution [75].

In addition to the clinical application of the m6A modifications described above, m6A methylation regulators have also been described as efficacious therapeutic targets for anticancer drugs in clinical treatment. Recently, studies have confirmed that therapeutically targeting METTL3 may offer an alternative to anti-vascular therapy for bladder cancer [56]. In addition, the m6A reader IGF2BP3 regulates cell cycle and angiogenesis in colon cancer. This study may provide a potential therapeutic target for colon cancer [39]. Furthermore, glioma cells become more sensitive to Taxol, temozolomide, and doxorubicin after the IGF2BP3 gene is knocked down, suggesting a role for IGF2BP3 in chemotherapy resistance [75].

In summary, m6A-related enzymes are not only used as markers of tumor prognosis but also act as drug targets to enhance the sensitivity of tumor treatments. Some clinical trials of RNA methylation in clinical applications are as follows (Table 2).

**Table 2:** Clinical trials of the RNA methylation in clinical applications. (Data source: United States clinical trial database https://beta.clinicaltrials.gov/)

Study title	Application phase	Disease	Clinical Trial No.
Oral Administration of STC-15 in Subjects With Advanced Malignancies	Phase 1	Advanced Cancer Advanced Solid Tumor	NCT05584111
The Red Blood Cells Based Blood Test for Lung Cancer EARLY Detection	Not Applicable	Lung Cancer	NCT05380999
Impact of 5-Fluorouracil (5-FU) on Cancer Cells From Liver Metastases of Colon Cancer (FLUORIB)	Not Applicable	Cancer of Colon Metastasis to Liver	NCT04274790
The Role of m6A RNA Modification in Moyamoya Disease	Not Applicable	Moyamoya Disease	NCT04696094

## Conclusions

In this review, we summarized the progress of research on the role of RNA modification in tumor angiogenesis. We evaluated the biological functions of RNA modification in altering drug resistance and activating signalling pathways. There are various pathways that are involved in this regulation, and they exist in a variety of cancers. In addition, a variety of miRNA molecules that are related to RNA methylation are also involved in tumor angiogenesis and tumor progression. RNA methylation also affects tumor invasion and the degree of vascular invasion by affecting the BBB. Furthermore, RNA methylation related molecules may be used as biomarkers to measure tumor progression and predict prognosis. More importantly, studying the effect of RNA methylation on tumor angiogenesis is beneficial for identifying novel therapeutic drugs to control cancer progression.

Despite the abovementioned encouraging advances, there are still multiple challenges and difficulties associated with the application of RNA methylation in clinical practice. It should be noticed that such applications focus on a single RNA modification and its corresponding regulators [171]. We need more systematic studies to investigate the relationship between RNA methylation and tumor angiogenesis. In addition, accumulating evidence has indicated that RNA modifying proteins((RMPs) has significant potential as pharmacological targets or diagnostic markers [172]. However, the interactions between different RNA modifications and modifiers are only just beginning to be elucidated, leaving some doubt about the clinical application of RMPs. To date, the clinical applications of METTL3 inhibitors have been systematically reviewed [173]. For example, STM2457, which is a highly potent and selective first-in-class catalytic inhibitor of METTL3, has been proven to decrease AML growth and increase differentiation and apoptosis [174]. However, not all of these inhibitors have demonstrated acceptable target potency or enzyme selectivity and the clinical applications of many other modifiers still require further research [175]. Additionally, the effects of RNA methylation on some cancers remain unknown.

In brief, RNA methylation plays an essential role in the regulation of tumor angiogenesis and cancer progression. Although RNA methylation shows great potential for clinical application, there is still substantial work to be done before it can be applied to clinical practice due to current technical limitations.

## Data Availability

Not applicable.

## Abbreviations

ALKBH: Alkb homologue
BBB: Blood–brain barrier
BCLC: Barcelona Clinic Liver Cancer
CBLL1: Cbl proto-oncogene like 1
CCR7: CC-chemokine receptor 7
CRC: Colorectal cancer
CTGF: Connective tissue growth factor
EC: Endothelial cell
EGFR: Epidermal growth factor receptor
EMT: Epithelial mesenchymal transition
ENO2: Enolase 2
FTO: Fat mass and obesity-associated protein
GBM: Glioblastoma
GC: Gastric cancer
GLUT4: Glucose transporter type 4
HCC: Hepatocellular carcinoma
HDGF: Hepatoma-derived growth factor
HIF: Hypoxia inducible factor
HMGA2: High mobility group A2
HNF4G: Hepatocyte nuclear factor 4 gamma
HNRNP: Heterogeneous nuclear ribonucleoprotein
ICC: Intrahepatic cholangiocarcinoma
IGF2BPs: Insulin growth factor-2 binding proteins
IL11: Interleukin 11
m5c: 5-methylcystosine
m6A: N6-methyladenosine
METTL: Methyltransferase-like
miRNA: MicroRNA
MTHFD2: Methylenetetrahydrofolate dehydrogenase 2
MVD: Microvessel density
PRMT5: Protein arginine methyltransferase 5
RBM: RNA-binding motif
RCC: Renal cell carcinoma
RTK: Receptor tyrosine kinase
SERPINE2: Serpin family E member 2
TAZ: Transcriptional co-activator with PDZ-binding motif
TK1: Thymidine kinase 1
TNFR: Tumor necrosis factor receptor
TNS1: Tensin1
TRAF: Tumor necrosis factor receptor-associated factor
VASH1: Vasohibin-1
VEGF: Vascular endothelial growth factor
VIRMA: Vir-like m6 A methyltransferase associated
VM: Vasculogenic mimicry
WTAP: Wilms Tumor 1 associated protein
YAP: Yes-associated protein
YTHDF: YT521-B homology domain-containing protein family
ZC3H13: Zinc finger CCCH-type containing 13
ZNRD1-AS1: Zinc ribbon domain-containing 1-antisense 1
